# Whole exome sequencing reveals two novel compound heterozygous mutations in *TWNK* as a cause of the hepatocerebral form of mitochondrial DNA depletion syndrome: a case report

**DOI:** 10.1186/s12881-019-0875-y

**Published:** 2019-08-27

**Authors:** Xianghong Li, Liangshan Li, Yaqi Sun, Fuyan Lv, Guoqing Zhang, Wenmiao Liu, Meiyan Zhang, Hong Jiang, Shiguo Liu

**Affiliations:** 1 Department of Neonatology, The Affiliated Hospital of Qingdao University, Qingdao, 266000 China; 2 Medical Genetic Department, The Affiliated Hospital of Qingdao University, Qingdao, 266000 China; 3 Qingdao Women and Children’s Hospital, Qingdao, 266034 China; 4 Department of Orthopaedic Surgery, the Affiliated Hospital of Qingdao University, Qingdao, 266000 China; 5 Shandong Provincial Key Laboratory of Metabolic Diseases and Qingdao Key Laboratory of Gout, The Affiliated Hospital of Qingdao University, Qingdao, 266000 China

**Keywords:** Hepatocerebral form of MDS, *TWNK*, Whole exome sequencing (WES), Sanger sequencing

## Abstract

**Background:**

Although Mitochondrial DNA depletion syndrome (MDS) can be classified into three forms: myopathic, encephalomyopathic and hepatocerebral form, it is difficult to identify its form due to its clinical heterogeneity. Therefore, it is very important to conduct molecular genetic analysis on suspected patients. This study presented a male 38 weeks and 5 days infant with liver cytolysis and leukodystrophy.

**Case presentation:**

A male infant proband was admitted to the department of NICU for feeding intolerance, irregular rhythm of respiration, hypoglycemia, lactic acidosis, liver cytolysis and neurological abnormalities. He was onset of mild jaundice with leukodystrophy and high lactate and phenylderivatives for urine organic acids on the 7th day. Whole exome sequencing (WES) and Sanger sequencing were performed to screen and confirm the suspicious pathogenic mutations. The results revealed this proband carried two compound heterozygous mutations in *TWNK*: c.1186 C > T / p.Pro396Ser and c.1844 G > C / p.Gly615Ala inherited by an autosomal recessive form from his parents, of which protein conservative analysis and structural modeling supported the pathogenicity of the two mutations. Unfortunately, the conditions described above were not improved until he was discharged from the hospital on the 23rd day and died at 4 months of age.

**Conclusions:**

In this study, we investigated a Chinese family with the hepatocerebral form of MDS and conducted WES and Sanger sequencing to explore the causative mutations for this proband born from non-consanguineous and healthy parents. We identified two novel *TWNK* c.1186 C > T/ c.1844 G > C compound heterozygous mutations which were probably the disease-causing mutations of hepatocerebral form of MDS and described the clinical manifestations of the proband, which expanded the phenotypic spectrum of MDS caused by variants in *TWNK*. This study also emphasized WES technology can provide the genetic diagnosis of Mendelian genetic disease.

## Background

The mitochondrial DNA (mtDNA) encodes 13 essential subunits of the respiratory chain complexes, 2 ribosomal RNA and 22 transfer RNA, whereas all the other components required for mitochondrial structure and function are encoded by the nuclear genome. Mutations in nuclear genes involved in the replication and maintenance of the mtDNA can result in the reduction of the mtDNA copy number, the accumulation of mtDNA multiple deletions, or both alterations in critical tissues [1]. Mitochondrial DNA depletion syndrome (MDS), characterized by the reduction of the mtDNA copy number and transmitted in an autosomal recessive trait, is clinically and genetically heterogeneous [2]. Insufficient synthesis of respiratory chain complexes may affect either a specific tissue or multiple organs such as liver, brain, muscle and kidney. MDS can be classified into three forms: myopathic, encephalomyopathic and hepatocerebral form according to clinical characteristics [3] and relevant different gene mutations. Genetically, mutations in thymidine kinase genes (*TK2*) [4] and ribonucleotide reductase regulatory TP53 inducible subunit M2B (*RRM2B*) [5] are responsible for myopathic form of MDS, while mutations of *SUCLA2* (succinate-CoA ligase ADP-forming beta subunit) [6] and *SUCLG1*(succinate-CoA ligase alpha subunit) [7] are related to encephalomyopathic form of MDS. In addition, twinkle mtDNA helicase (*TWNK,* OMIM # 606075), DNA polymerase gamma, catalytic subunit (*POLG*) [8, 9], the deoxyguanosine kinase (*DGUOK*) [10] and the recently stated mitochondrial inner membrane protein (*MPV17*) [11] are the most common candidate genes of hepatocerebral form. Due to the complex clinical manifestations of mitochondrial disorders, whole exome sequencing (WES) followed by Sanger sequencing was performed to provide the genetic diagnosis of MDS.

Hepatocerebral form of MDS identified in children between birth and six months old presents multisystem phenotypes including liver failure, hypoglycemia, hypotonia and neurological symptoms, and patients usually die within one year of age [12] . Indeed, mutations in *TWNK* with 5 exons mapped to 10q24.31 can bring about hepatocerebral form of MDS with characteristic enlargement of liver and spleen, damage of liver function, retardation of psychomotor development, nystagmus and muscle hypotonia [13–15]. Twinkle, encoded by *TWNK* and located in the mitochondrial matrix and mitochondrial nucleoids, plays an important role in mtDNA replication [16]. *TWNK* was also associated with autosomal dominant progressive external ophthalmoplegia (adPEO, OMIM #609286) which can result in ophthalmoparesis with exercise intolerance, ataxia, peripheral neuropathy and multiple mtDNA deletions and at least 40 causative mutations for adPEO have been identified up to now [17]. Besides, several cases of *TWNK* mutations in an autosomal recessive inherited trait can cause infantile onset spinocerebellar ataxia (IOSCA) (OMIM #271245) with the main clinical features including muscle hypotonia, athetosis, ataxiaophthalmoplegia, hearing deficit, sensoryaxonal neuropathy, female hypogonadism and epilepticencephalopathy [18] and Perrault syndrome 5 (PRLTS5, OMIM #616138) with hearing loss, female hypergonadotropic hypogonadism and ataxia [17].

Here, we described an infant proband born in a Chinese non-consanguineous family with clinical features of hepatocerebral form of MDS and identified novel compound heterozygous variants in *TWNK*.

## Case presentation

### Materials and methods

#### Subjects

This study investigated an infant proband with hepatocerebral form of MDS from a Chinese family. However, his parent didn’t exhibit similar symptoms of the disease. Written informed consent was obtained from the patient according to the protocol approved by the Institutional Review Board of The Affiliated Hospital of Qingdao University.

#### Wes

WES technology was performed to screen the mutational sites. Genomic DNA of the proband and his parent were extracted from the peripheral blood using the QIAamp DNA Blood Mini Kit (Qiagen, Hilden, Germany). The DNA samples were prepared as Illumina sequencing libraries. The sequencing libraries were constructed with SureSelect Human All Exon V5 (Agilent Technologies, Santa Clara, CA), according to the manufacturer’s protocols and were sequenced using Hiseq 4000 next-generation sequencing platform (Illumina, San Diago, CA). Paired-end sequence reads were aligned to the human reference genome hg19 with Burrows-Wheeler Aligner (BWA), and then the variant calling process was run using the Genome Analysis Tool Kit (GATK). Variants were submitted to ANNOVAR for functional annotation and genetic filtering. Common variants were excluded by comparison with > 1000 exomes sequenced in our laboratory for other conditions and subsequently filtered with dbSNP v137, 1000 Genomes Project, National Heart, Lung, and Blood Institute (NHLBI), Exome Sequencing Project (ESP) and ExAC databases. Finally, functional impacts of the variants were predicted in silico by corrsponding softwares. Missense mutations were analyzed by PolyPhen-2 (http://genetics.bwh.harvard.edu/pph2/) and SIFT (http://sift.bii.a-star.edu.sg) whereas evolutionary conservation of the missense mutation sites were analyzed by aligning Twinkle amino-acid sequences of multiple species obtained from the UniProt website (http://www.uniprot.org).

#### Sanger sequencing

The variants detected by WES were validated using Sanger sequencing. Amplifications were performed with primers targeting the variant loci as follows: forward, 5′-CAGTTCCGGCGGATTGTATT-3’and reverse, 5′-GTCCTACCCTTGAAACCAAGACA-3’for c.1186 C > T in *TWNK* and forward, 5′-AGCCCCTCTCCCCATTCTTA-3’and reverse, 5′-GAGCCCTTGCAGAGTTTTATGC-3’for c.1844 G > C in *TWNK*. The polymerase chain reaction was conducted by following conditions: initial denaturation; 35 cycles of denaturation, annealing and extension; the final extension. The PCR products were purified and subsequently sequenced using Genetic Analyzer 3130 capillary sequencer (Applied Biosystems).

#### Protein structure modeling

The structure of T7 bacteriophage primase-helicase gp4 (PDB 1e0kA), a Twinkle ortholog, was used to predict the structural roles of Twinkle residues [13]. Comparative modeling methods and energy minimization were applied to model the three-dimensional structure of Twinkle by SWISS-MODEL program [19]. Orthologous residues were determined by amino acid sequence alignment with ClustalW-2. Structures were visualized and cartoons rendered using the PyMOL.

## Results

### Clinical presentation

The proband was a male infant, born in 38 weeks and 5 days with a weight of 2950 g (10th centile). Apgar scores were 10 at 1, 5 and 10 min. He presented feeding intolerance, irregular rhythm of respiration, hypoglycemia (peripheral blood glucose: 1.2 mmol/L, Normal> 2.2 mmol/L), lactic acidosis (lactate: 9.6 mmol/L, Normal < 2.8 mmol/L, artery blood PH 7.2, Normal = 7.35~7.45, PCO_2_ 18 mmHg, Normal = 35~48 mmHg, buffuer excess − 18.6 mmol/L, Normal = − 2~ − 3 mmHg), liver cytolysis (aspartate aminotransferase: 345.5 UI/L, Normal = 15~40 UI/L; alanine aminotransferase: 82.9 UI/L, Normal = 7~40 UI/L; γ-glutamyltranspeptidase: 574 UI/L, Normal = 6~25 UI/L) and neurological abnormalities including: hyperspasmia, peripheral hypertonia and no primitive reflex. Biochemical investigations showed high plasma creatine kinase: 1360.6 UI/L (Normal = 50~310 UI/L), high isoenzyme of creatine kinase concentration:177 UI/L(Normal = 0~17 UI/L), high lactate dehydrogenase (1261.2 UI/L, Normal = 120~250 UI/L), high plasma ammonia: 90 μmol/L(Normal = 9~33 μmol/L), high uric acid: 510 μmol/L(Normal = 89.2~416 μmol/L) and high blood urine nitrogen:10.31 mmol/L(Normal = 3.1~8 mmol/L), while blood and body fluids showed no evidence of infection. In addition, there was elevated level of plasma tyrosine:1054 μmol/L (Normal = 25~250 μmol/L) without associated evaluations in urinary succinylacetone. Urine organic acids revealed high lactate and phenylderivatives. Brain MRI showed leukodystrophy. The mild jaundice was noted (total bilrubin: 250.79 μmol/L, Normal = 3~22 μmol/L, direct bilirubin: 52.20 μmol/L, Normal = 0~8 μmol/L) on the 7th day. The clinical manifestations were not get better until he left the hospital on the 23rd day and died at 4 months old.

### Identification of the novel mutations in *TWNK*

**Fig. 1:**
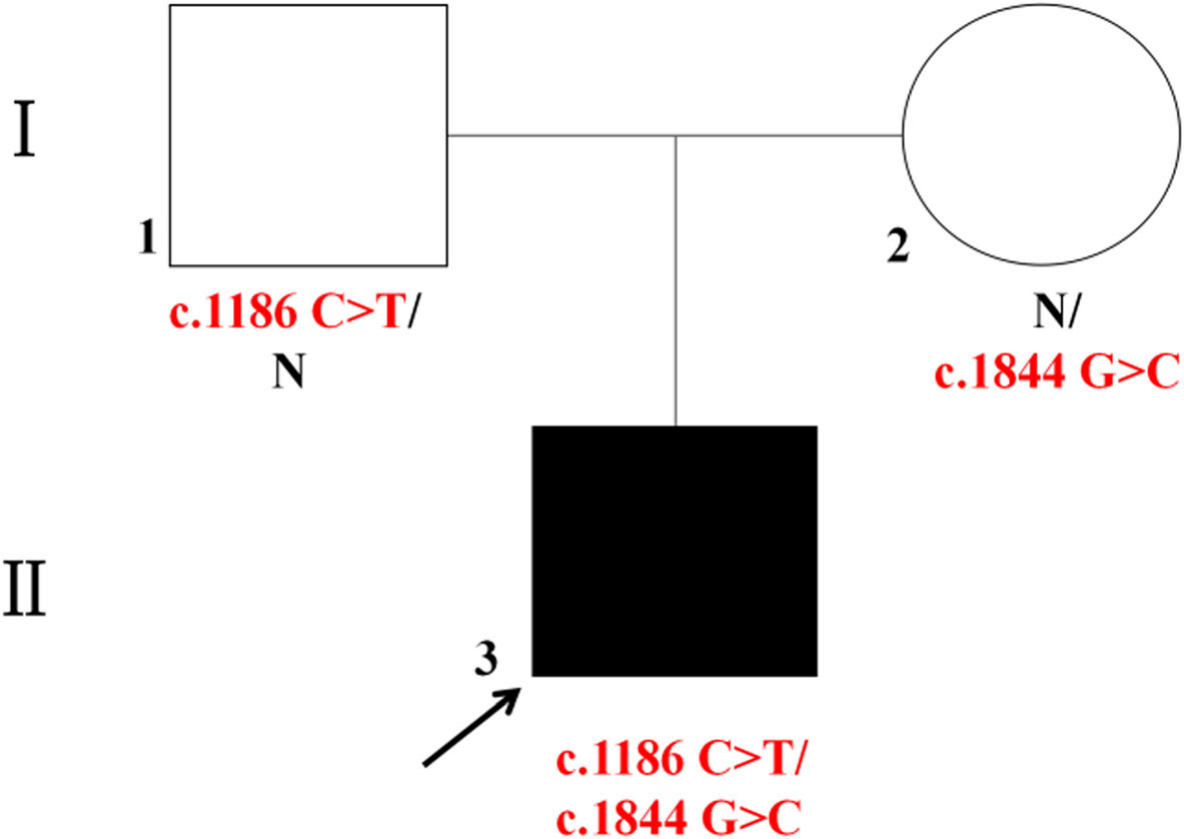
Pedigree of *TWNK* mutations in this family with MDS (Filled symbols indicate affected individuals; arrowhead indicates the proband)

**Fig. 2:**
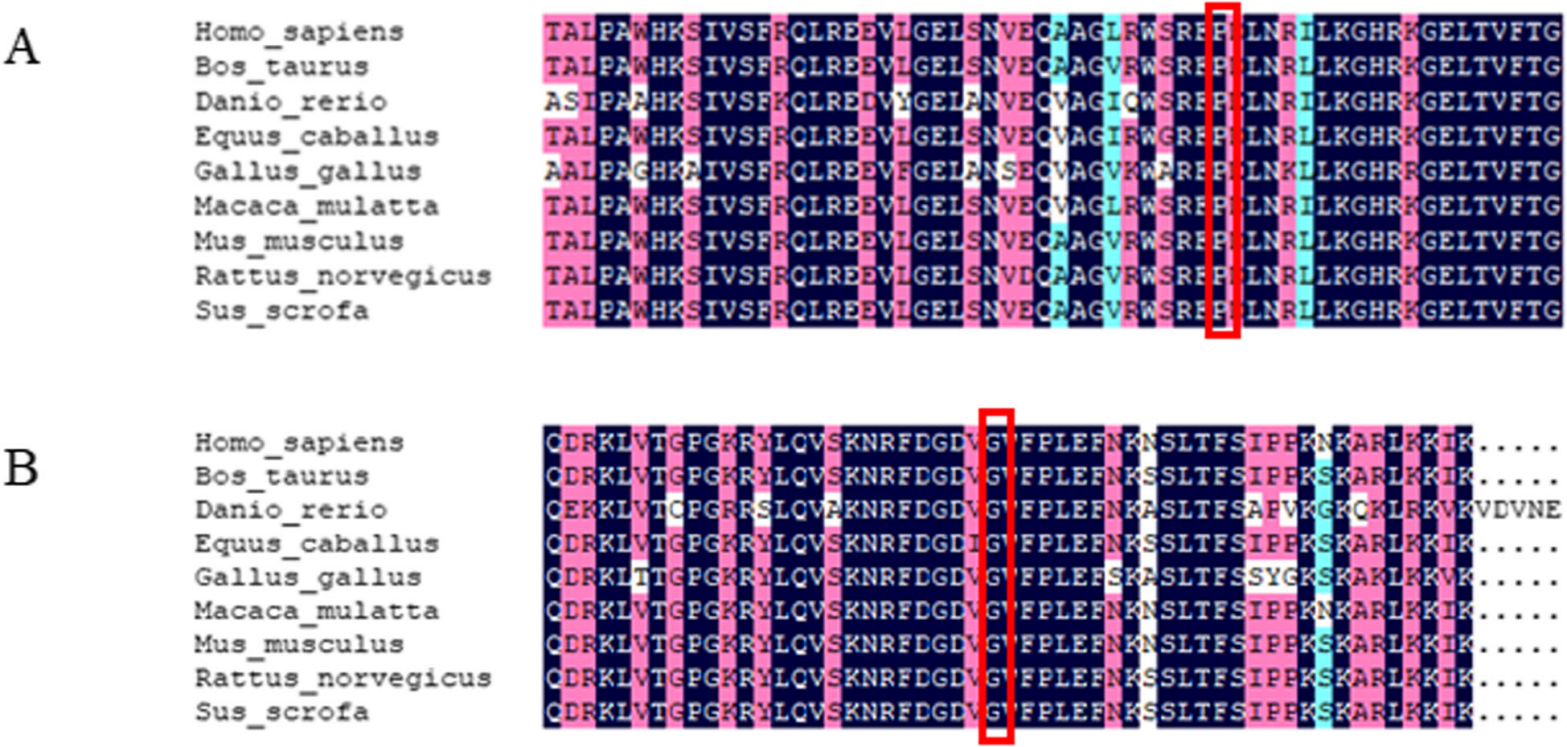
Conservation of protein sequence at 2 residues with mutations in this family (Mutated residues are framed in red). (**a**) Conservation of p.Pro396Ser (**b**) Conservation of p.Gly615Ala

**Fig. 3:**
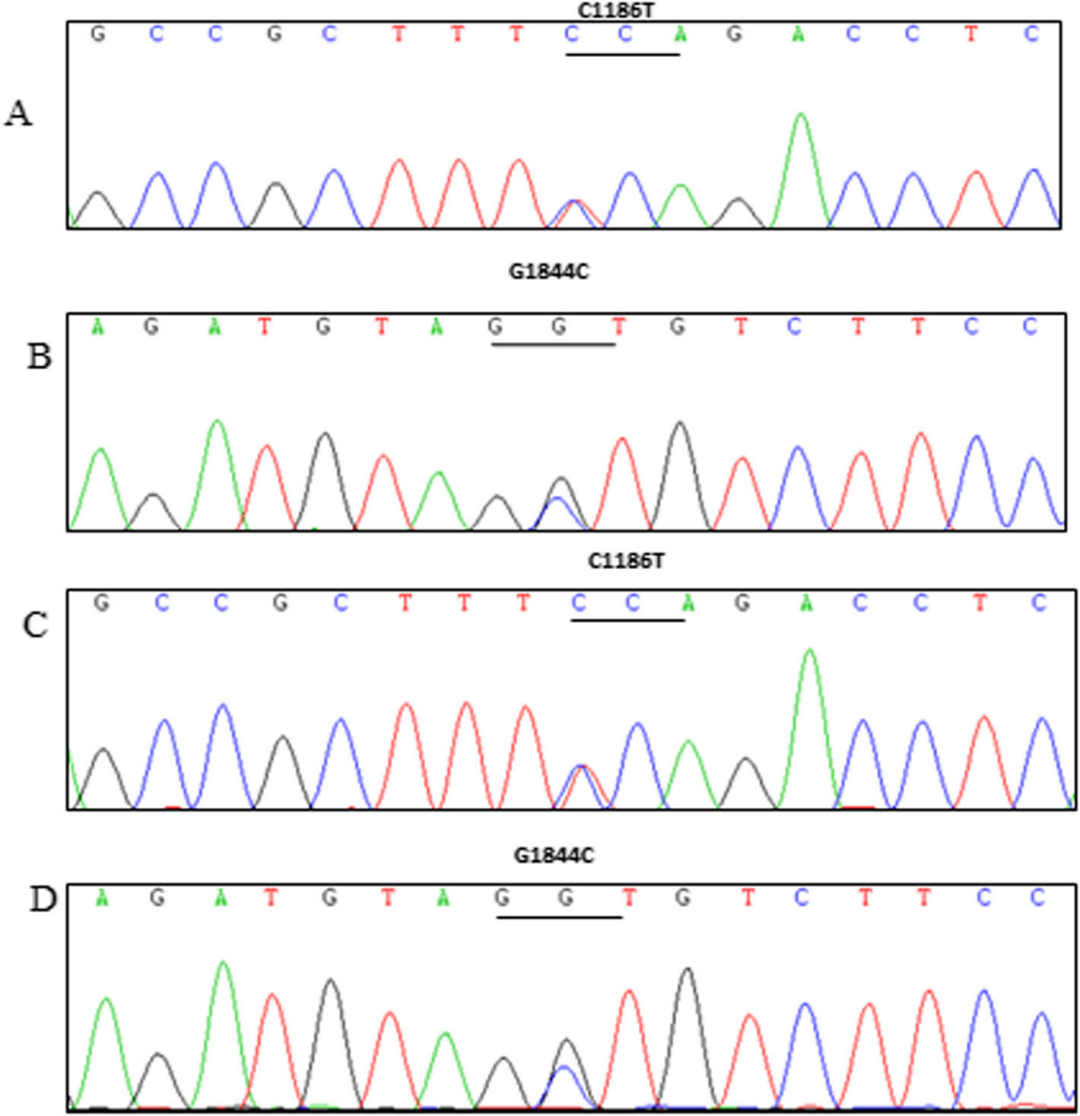
Sequence chromatograms of this Chinese family. (**a**) *TWNK* c.1186C > T (p.Pro396Ser) from the proband (**b**) *TWNK* c.1844G > C (p.Gly615Ala) from the proband (**c**) *TWNK* c.1186C > T (p.Pro396Ser) from the father (**d**) *TWNK* c.1844G > C (p.Gly615Ala) from the mother

We performed WES combined with Sanger sequencing to identify disease-causing mutations in this Chinese family affected by hepatocerebral form of MDS. After the results were excluded in comparison with > 1000 exomes sequenced in our laboratory and filtered with the frequencies of dbSNP v137, 1000 Genomes Project, National Heart, Lung, and Blood Institute (NHLBI), Exome Sequencing Project (ESP) and ExAC databases, we identified potentially shared homozygous or compound heterozygous potentially damaging variants. We found the proband carried two compound heterozygous mutations in *TWNK* (c.1186 C > T / p.Pro396Ser in exon 1, inherited from the father, and c.1844 G > C / p.Gly615Ala in exon 5, inherited from the mother) (Fig. 1). Both mutations were not described associated with hepatocerebral form of MDS before. CADD (score 3.361291)and CADD Phred(score 17.33) predicted that it was benign for c.1186 C > T, however, PolyPhen-2 (score 0.716), SIFT (score 0.02)and Provean (score − 6.88) revealed an opposite result that the mutation c.1186 C > T was deleterious. The c.1844 G > C heterozygous variant was predicted to be damaging by PolyPhen-2 (score 1.00), SIFT (score 0.00), CADD (score 5.129582), CADD Phred(score 32)and Provean (score − 5.46). Protein multiple sequence alignment of Twinkle suggested that both variants were located at the highly conserved regions of the Twinkle protein (Fig. 2a, b). The compound heterozygous mutations were not found in 200 normal chromosomes from 100 healthy controls of Chinese Han origin and co-segregation analysis in this pedigree through Sanger sequencing demonstrated that the two missense mutations were likely the pathogenic variants responsible for the MDS clinical manifestations of this proband (Fig. 3).

### Twinkle modeling

**Fig. 4:**
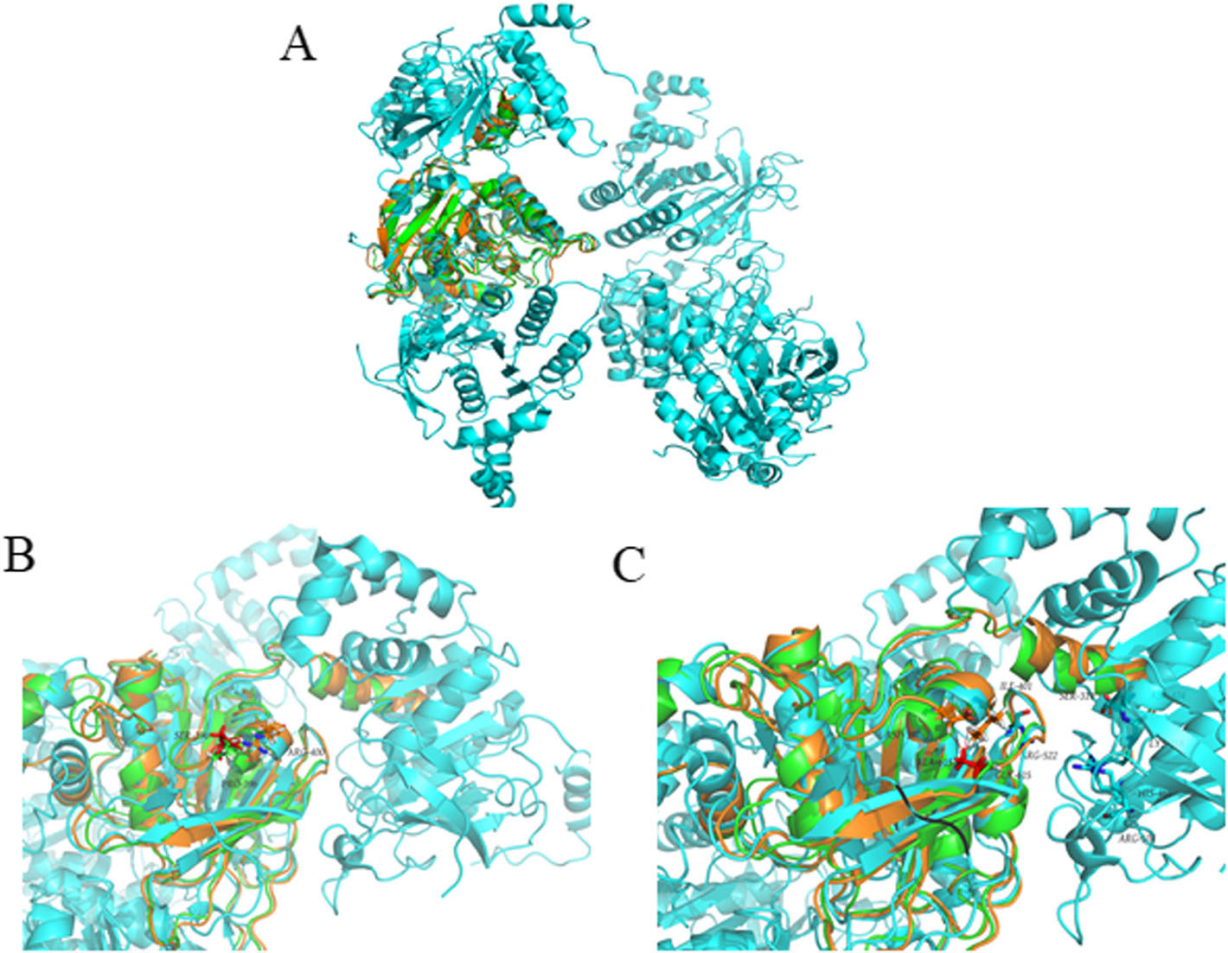
Model of the three-dimensional structure of Twinkle protein (The homohexameric T7 gp4 helicase domains are in cyan). (**a**) The modeled protein green and superimposed with the blue subunit. Mutated protein is in orange. (**b**) Magnification of the the linker domain where Pro396 is localized at. Mutated Ser396 is in red. (**c**) Magnification of the the linker domain where Gly615 is localized at. Mutated Ala 615 is in red

With highest similarity scores obtained for the helicase domain of Twinkle (residues 367–623, 24% identity, 41% similarity), gp4 primase/helicase from phage T7 (PDB code 1e0kA) was used to model the possible structure of the *TWNK* mutations in the proband. The contributed structure was energy minimized by Amber and shown as PyMOL Viewer representation, demonstrating that the two structures were similar (Fig. 4). Twinkle consists of three functional domains: an N-terminal primase, a linker region involved in oligomerization and required for helicase activity and a C-terminal helicase [20]. The gp4 primase/helicase from phage T7 and the modeled Twinkle protein showed that Proline at position 396 is localized at the linker domain. The p.Pro396Ser substitution may alter the flexible of the linker by formation of hydrogen bonds with Arg400, thus interfer the interaction of the linker. Glycine at the 615th position of Twinkle is localized at the helicase domain that it has the function of binding with nucleotides. The NTP binding pocket of helicase is located between two subunits. The subtitution of alanine for glycine shortens its distance to Asp397 and Ile401, which may make the two subunits become too close through Van der Waals force. Arg552 locates at the top of one subunits. When the two subunits get closer, Arg552 may extrude the NTP binding pocket, thus the NTP binding pocket cannot bind NTP because of its smaller space.

## Discussion and conclusions

MDS may affect either a specific tissue (especially muscle, liver and brain) or multisystem including heart, brain and kidney. Clinically, MDS were classified into three forms: myopathic, encephalomyopathic and hepatocerebral form [21]. Due to complex clinical manifestations of MDS, it is difficult to distinguish its type and it is very important to perform molecular genetic analysis on suspected patients. In this study, we conducted WES together with Sanger sequencing validation on the proband with MDS and Confirmed two novel compound heterozygous mutations in *TWNK*: c.1186 C > T / p.Pro396Ser in exon 1 and c.1844 G > C / p.Gly615Ala in exon 5, which were transmitted in a autosomal recessive trait. The former was inherited from the father while the later was from the mother, both of which were not described associated with mitochondrial disorders before.

Twinkle, encoded by *TWNK*, plays an important role in the integrity and maintenance of the mitochondrial DNA and displays 5′ → 3′ DNA helicase activity in unwinding the mitochondrial DNA at replication fork [22]. Autosomal recessive inheritance conditions caused by mutations in *TWNK* have been summarized as Perrault syndrome 5(OMIM #616138) and Mitochondrial DNA depletion syndrome 7 (MTDPS7, OMIM # 271245). The main clinical features of Perrault syndrome 5 are hearing loss, female hypergonadotropic hypogonadism and ataxia [23], however the various phenotypes of MTDPS7 including IOSCA characterized by the development of musclehypotonia, ataxia, ophthalmoplegia, optic atrophy, hearing loss, sensory axonal neuropathy,epilepsy, and female hypogonadism about first of the year [18, 24] and the more severe hepatocerebral form of MDS involving liver failure and neurological abnormalities after birth [14, 15, 25, 26]. The clinical manifestations of this proband described here were similar to the hepatocerebral form of MDS, which presented as born with hepatic failure, progressive neurological manifestations and severe clinical symptoms including: a feeding intolerance, irregular rhythm of respiration, hypoglycemia accompanied by lactic acidosis, elevation of plasma tyrosine, ammonia, uric acid and blood urine nitrogen. This proband developed multisystem phenotypes when he was newborn whereas the age of onset in IOSCA is on the average only 14 months and Perrault syndrome is even later at 6 or 7 years old. Besides, the severe multisystem phenotypes including liver failure could exclude the IOSCA and Perrault syndrome 5.

Up to now, a total of 13 *TWNK* mutations leading to MTDPS7 have been identified, with four of these various causing the severe hepatocerebral form of MDS. Compound heterozygous *TWNK* mutations (p.A318T and p.Y508C) were found in two siblings with severe encephalopathy and signs of liver involvement at 6 months old. The clinical manifestations included hypotonia, athetosis, sensory neuropathy, ataxia, hearing deficit, ophthalmoplegia, intractable epilepsy and elevation of serum transaminases [15]. p.T457I mutation in *TWNK* in two patients presented neurological abnormalities, liver enlargement and failure, lactic acidosis at birth and abnormal eye movements [13]. Except for typical symptoms, patients with p.R29∗/p.Y508C compound heterozygous mutations had specific clinical manifestations, such as dysconjugate gaze, ophthalmic signs including optic atrophy, delayed or absent visual-evoked potentials and seizure activity [26]. In 2013, Prasadet elucidated p.F395 L homozygous mutation in *TWNK* as the cause of multisystem failure including renal tubulopathy in three siblings [14]. In our study, besides typical neurological abnormalities and liver failure, some biochemical examination results firstly described in hepatocerebral form of MDS comprise of elevated plasma creatine kinase and isoenzyme of creatine kinase concentration with 10 times the upper limit of normal. High lactate dehydrogenase: 1261.2 UI/L (Normal = 120~250 UI/L), high plasma ammonia: 90 μmol/L (Normal = 9~33 μmol/L), high uric acid: 510 μmol/L (Normal = 89.2~416 μmol/L) and leukodystrophy also expand the phenotypic spectrum of MDS which is associated with mutated *TWNK*.

Three functional domains forms the Twinkle: an N-terminal primase, a linker region involved in oligomerization and required for helicase activity and a C-terminal helicase [20]. In this case, the structure modeling and biochemical analysis of the p.Pro396Ser indicated that the substitution may alter the flexible of the linker by formation of hydrogen bonds with Arg400, thus interfere the interaction of the linker. Gly615, localized at the helicase domain, has the function of binding with nucleotides. As the NTP binding pocket of helicase is located between two subunits, The substitution of alanine for glycine at position 615 makes the two subunits become too close. As for Arg552, located at the top of one subunit, it may extrude the NTP binding pocket, thus disrupt the coupling between NTP hydrolysis and single stranded DNA binding.

Further involving multiple systems makes MDS exhibit a variety of clinical phenotypes, however the heterogeneity of clinical features of this disease makes it challenging to reach a definitive diagnosis in this case. By performing WES and Sanger sequencing, we identified two novel *TWNK* mutations which are probably pathogenic mutations of hepatocerebral form of MDS. Our study emphasizes the utility of WES for the genetic diagnosis of complex multisystem disorders. Due to the diverse clinical manifestations of mitochondrial disorders, identification of causive allele by WES is now the gold standard for evaluation.

## Data Availability

The data and materials used and analysed during the current study are available from the corresponding author upon reasonable request.

## Abbreviations

adPEO: Autosomal dominant progressive external ophthalmoplegia
BWA: Burrows-Wheeler Aligner
DGUOK: Deoxyguanosine kinase
ESP: Exome Sequencing Project
GATK: Genome Analysis Tool Kit
IOSCA: Infantile onset spinocerebellar ataxia
MDS: Mitochondrial DNA depletion syndrome
MPV17: Mitochondrial inner membrane protein
MRI: Magnetic Resonance Imaging
MTDPS7: Mitochondrial DNA depletion syndrome 7
NHLBI: National Heart, Lung, and Blood Institute
NICU: Neonatal Intensive Care Unit
PDB 1e0Ka: T7 bacteriophage primase-helicase gp4
POLG: DNA polymerase gamma, catalytic subunit
RRM2B: Ribonucleotide reductase regulatory TP53 inducible subunit M2B
SUCLA2: Succinate-CoA ligase ADP-forming beta subunit
SUCLG1: Succinate-CoA ligase alpha subunit
TK2: Thymidine kinase 2
TP53: Tumor protein p53
